# Sniffing Like a Wine Taster: Multiple Overlapping Sniffs (MOSS) Strategy Enhances Electronic Nose Odor Recognition Capability

**DOI:** 10.1002/advs.202305639

**Published:** 2023-12-14

**Authors:** Luzheng Liu, Na Na, Jichuan Yu, Wenxiang Zhao, Ze Wang, Yu Zhu, Chuxiong Hu

**Affiliations:** ^1^ State Key Laboratory of Tribology Department of Mechanical Engineering Tsinghua University Beijing 100084 China; ^2^ Key Laboratory of Radiopharmaceuticals Ministry of Education College of Chemistry Beijing Normal University Beijing 100875 China

**Keywords:** active sniff, bionics, computational fluid dynamics, electronic nose, gas detection, liquor recognition

## Abstract

As highly promising devices for odor recognition, current electronic noses are still not comparable to human olfaction due to the significant disparity in the number of gas sensors versus human olfactory receptors. Inspired by the sniffing skills of wine tasters to achieve better odor perception, a multiple overlapping sniffs (MOSS) strategy is proposed in this study. The MOSS strategy involves rapid and continuous inhalation of odorants to stimulate the sensor array to generate feature‐rich temporal signals. Computational fluid dynamics simulations are performed to reveal the mechanism of complex dynamic flows affecting transient responses. The proposed strategy shows over 95% accuracy in the recognition experiments of three gaseous alkanes and six liquors. Results demonstrate that the MOSS strategy can accurately and easily recognize odors with a limited sensor number. The proposed strategy has potential applications in various odor recognition scenarios, such as medical diagnosis, food quality assessment, and environmental surveillance.

## Graphical abstract

Inspired by expert wine tasters, a multiple overlapping sniffs (MOSS) strategy is presented to enhance electronic noses' odor detection. By employing rapid, successive inhalations of odorants, this strategy elicits rich temporal signals from sensor arrays. MOSS strategy achieves over 95% accuracy in recognizing gaseous alkanes and liquors, holding promise for odor recognition in medical, food, and environmental applications.

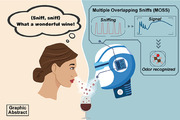

## 1 Introduction

Olfaction, the sense of smell, plays a critical role in ensuring the survival, well‐being, and enjoyment of human beings. In recent years, there has been a surge of interest in questing electronic noses capable of matching or even surpassing human olfaction.^[^ ^1^ ^]^ An electronic nose typically has a sensor array that can cross‐respond to odorants, akin to human olfactory receptors (OR). Subsequently, pattern recognition algorithms are employed on the electronic nose to process these cross‐response signals and recognize odors.^[^ ^2^ ^]^ To date, electronic noses have exhibited promising potential across diverse fields of odor discrimination, including but not limited to medical diagnosis,^[^ ^3^, ^4^ ^]^ food quality assessment,^[^ ^5^ ^]^ precision agriculture,^[^ ^6^ ^]^ environmental surveillance,^[^ ^7^, ^8^ ^]^ and explosive detection.^[^ ^9^, ^10^ ^]^ Despite surpassing human olfaction in sensitivity and toxic gas detection capabilities, electronic noses are constrained by current sensor technology and still face challenges such as memorizing extensive odor libraries^[^ ^11^ ^]^ and recognizing complex odors.^[^ ^12^ ^]^

For the further performance enhancement of electronic noses, researchers have long been dedicated to constructing superior gas sensor arrays. A diverse range of sensing materials has been cultivated for application in various gas sensing scenarios, encompassing metal oxide semiconductors, carbon‐based materials, self‐assembled nanomaterials, etc.^[^ ^13^, ^14^ ^]^ Furthermore, leveraging machine learning algorithms, sensing materials can undergo targeted screening to attain more precise recognition ability tailored to specific aromas.^[^ ^15^ ^]^ On another front, progress in data processing algorithms is bolstering the recognition capabilities of electronic noses.^[^ ^16^ ^]^ For instance, data‐driven analyses such as principal component analysis (PCA), hierarchical cluster analysis (HCA), support vector machines (SVM), and their enhanced variants are extensively employed in electronic noses.^[^ ^17^ ^]^ With the development of deep learning, event‐driven artificial intelligence (AI) techniques such as recurrent neural networks (RNN) and convolutional neural networks (CNN) are involved in processing abundant gas sensing data of electronic noses and achieving higher performance in complex odor recognition tasks.^[^ ^18^ ^]^ Nowadays, bio‐inspired strategies for enhancing the sensitivity and selectivity of electronic noses have attracted the attention of researchers.^[^ ^19^ ^]^ For example, more selective sensitive sensing materials are designed based on biological proteins and bio‐probes to mimic olfactory receptor structure.^[^ ^20^ ^]^ Moreover, neuromorphic data processing techniques that mimic olfactory bulb cells have demonstrated commendable recognition capabilities with limitations of time and cost.^[^ ^21^ ^]^ To be clear, a biological nose is much more intricate than a sensor array. The binding of gas molecules to olfactory receptors occurs as gas flows through the nasal cavity during sniffing.^[^ ^22^ ^]^ Meanwhile, studies have proven that properly simulating the structure of canine nasal cavities and turbinate bones can improve the sensing speed and sensitivity of electronic noses.^[^ ^23^, ^24^ ^]^

In addition, sniffing is a common perceptual behavior, defined as the active sampling of odorants through the nasal cavity in order to acquire odor information.^[^ ^25^, ^26^ ^]^ Terrestrial vertebrates perform sniffs by regulating the depth and frequency of inhalations during which odorants flow dynamically in the nasal cavity and bind to olfactory receptors to stimulate olfactory signals.^[^ ^27^, ^28^ ^]^ Moreover, terrestrial vertebrates tend to adjust their sniffing strategies to meet behavioral demands in real time.^[^ ^29^ ^]^ For example, a rodent with a resting sniff frequency of 1–3Hz will instantly increase its sniff frequency to 12Hz upon introduction of odorants.^[^ ^30^, ^31^ ^]^ Similarly, experiments have demonstrated that high‐frequency sniffs can enhance human olfactory event‐related potentials.^[^ ^32^ ^]^ For certain occupations, such as wine tasters, gourmet chefs, perfumers and firefighters, the conscious use of different sniffing strategies is essential to enhance olfactory perception.^[^ ^33^, ^34^ ^]^ Meanwhile, the transient response of sensors under dynamic airflow has become an important research direction in electronic nose data processing.^[^ ^35^, ^36^ ^]^ Therefore, changes in sniffing strategies are important for vertebrate olfaction and have great potential for biomimetic applications.

The sniffing of electronic noses modulates the dynamic flow through the sensor surface and has proven effective on many types of sensing materials. For instance, Szczurek et al. proposed induced temporary disturbance of gas flow as “artificial sniffing” to affect the exposure conditions of metal‐oxide (MOX) sensors, and demonstrated that the sensor transient responses to temporary disturbance were discriminative of different volatile organic compounds (VOCs).^[^ ^37^ ^]^ Spencer et al. compared the sniff strategy of different mammals and proposed a MOX sensor system using sniffs to speed up gas detection.^[^ ^38^ ^]^ Furthermore, Ziyatdinov et al. applied a mechanical ventilator to simulate the biological sniffs with a steady respiration frequency.^[^ ^39^ ^]^ The MOX sensor array can more quickly identify the composition of acetone‐ethanol binary mixtures by extracting high frequency features. Moreover, Gaku et al. developed a membrane‐type surface stress (MSS) sensor array and a sniffing‐like cyclic measurement strategy.^[^ ^40^ ^]^ The transfer functions of MSS responses were calculated to recognize the odors of four liquids. In short, the sniff strategies have proven useful on several types of gas sensors, and the mechanism by which dynamic flow affects the transient response of sensors deserves further exploration. However, the response time of gas sensors used in existing studies is quite long, that is, more than 10 s, so that the high‐frequency features of their signals are significantly less compared to the low‐frequency features. Consequently, the performance improvement of electronic noses under sniff strategies is still not notable.

Recently, cataluminescence (CTL) has gained attention as a novel gas sensing principle in which VOCs are oxidized on the catalyst surface and excited to emit weak luminescence for detection.^[^ ^41^ ^]^ CTL has been developed for decades of years, exhibiting advantages such as high sensitivity, rapid response and simple instrumentation.^[^ ^42^ ^]^ In fact, CTL sensors typically achieve response and recovery times of less than 5 s, significantly faster than conventional gas sensors.^[^ ^43^ ^]^ This swiftness positions CTL sensors as an excellent platform to study sniff strategies at this stage.

In this paper, a multiple overlapping sniffs (MOSS) strategy is investigated to enhance the odor recognition capability of a CTL‐based electronic nose. With multiple sniffs in rapid succession, MOSS strategy can obtain multiple consecutive peaks with high resolution, while the response signals are obviously accumulated due to the overlap between sniffs. Therefore, the odor recognition can be carried out by extracting the features of signal peaks. First, to study the mechanism of MOSS strategy, computational fluid dynamics simulations are performed, which suggests that the cumulative effect of gas concentration and velocity leads to the rich temporal features of signals. Then, it is experimentally demonstrated that the single CTL sensor assisted by MOSS strategy can directly recognize three VOC gases, that is, ethylene, acetylene and butane, even when the gas concentrations are variable. Finally, experiments are preformed to compare the recognition performance of the four‐sensor electronic nose on six kinds of liquors. Notably, the recognition accuracy with the traditional measurement strategy is only 81.94%, while that with MOSS strategy is up to 95.83%. In short, our proposed strategy is based on dynamic airflow modulation and can be applied to improve the recognition capability of various electronic noses. This MOSS strategy is expected to be applied to a variety of odor recognition scenarios such as food industry, clinical diagnosis, explosive detection, etc.

## 2 Result

### 2.1 MOSS Strategy

**Figure 1:**
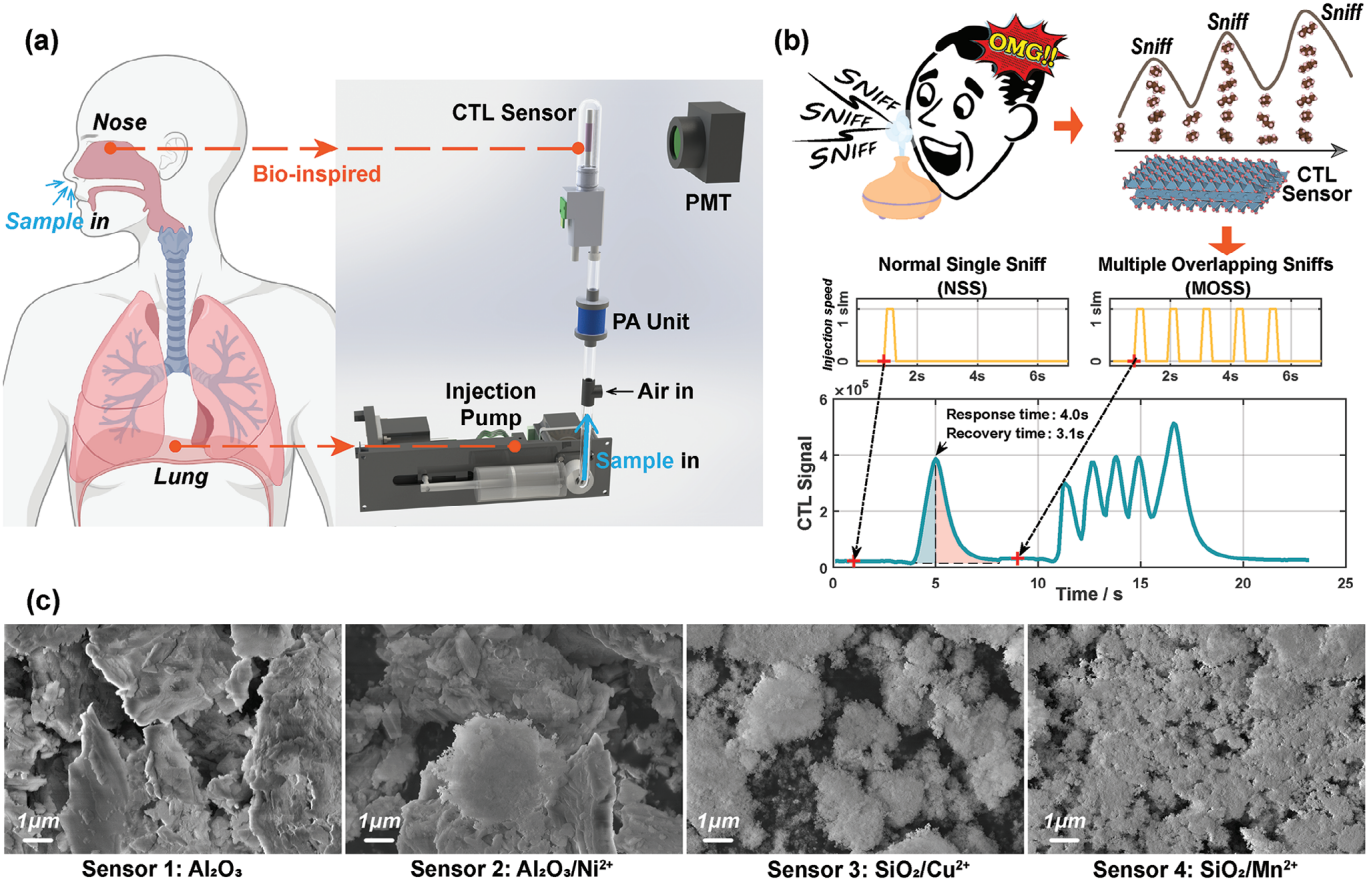
The electronic nose and its multiple overlapping sniffs (MOSS) strategy. a) Inspired by human olfaction, a CTL sensor array and an injection pump are used as an electronic nose and bionic lung to dynamically detect odors. b) MOSS strategy mimics vertebrate high‐frequency sniffs and applies consecutive sniffs to generate dynamic gas flow. Thanks to the fast response of CTL sensors, MOSS strategy can generate feature‐rich temporal signals. c) SEM images of four CTL sensor catalysts indicate the differences in their catalytic properties.

An electronic nose device with a bionic lung apparatus is meticulously fabricated (**Figure** **1**a). An injection pump serves as the bionic lung module, possessing an intake port and an exhaust port within a piston chamber. The injection pump has the capability to draw in gas from collected samples and deliver it into the subsequent conduit, ultimately reaching the sensor surface. Besides, its operational process is programmable, enabling the utilization of various sniffing strategies. It should be noted that the experimental device employs a unidirectional gas flow system, and CTL sensors possess distinct inlet and outlet ports, in contrast to the single entry and exit points of human nostrils. Therefore, the bionic lung module currently emulates inhalation but not exhalation. This structure is conducive to fast response and recovery of CTL sensors. Concurrently, the electronic nose incorporates four cataluminescence (CTL) sensors. A photomultiplier tube (PMT) is applied to capture the luminescence emitted by the CTL sensors. Furthermore, four distinct types of ion‐doped metal oxide nanoparticles are utilized as the catalysts of CTL sensors (Figure 1c). The employment of disparate catalysts (Figures S1 and S2, Supporting Information) engenders the capacity for these four CTL sensors to evince cross‐reactive responses to diverse gas samples, thus emulating the discerning behavior of natural olfactory receptors. Besides, the electronic nose device is equipped with a plasma‐assisted (PA) unit to increase the CTL sensor signal amplitude^[^ ^44^ ^]^ and a stable air input to improve the response speed of CTL sensors.^[^ ^45^ ^]^ Equipment dimensions are described in Experimental Section.

The conventional sampling strategy of electronic noses entails a singular introduction of the measured gas into the sensor array, yielding the response signals. This approach can be designated as a “normal single sniff” (NSS), delineated from a bionic perspective. The impediment constraining the advancement of intricate sampling methodologies resides in the sluggishness of sensor response time which curtails the capability of gas sensors to discriminate between dynamic gas flows. In contemporary times, however, a growing number of gas sensors are showing faster response times than ever before. In particular, the CTL sensors can exhibit response times of under 5 s, thereby facilitating an enhanced dynamic performance that allows for a more precise perception of the dynamic flow of gases. In light of this, a multiple overlapping sniffs (MOSS) strategy is proposed with the intention of delving further into sensor transient responses. The MOSS strategy involves the rapid, iterative inhalation of measured gas through the bionic lung (Figure 1b) at a consistent frequency. The sniffing interval, slightly shorter than the sensor response time, ensures that the response peaks corresponding to each inhalation remain independent yet slightly intersecting, resulting in plentiful temporal features including multiple continuous peaks and a distinctive tail peak. In fact, the signals serve as manifestations of the perpetually evolving dynamic airflow occurring on the sensor surface throughout the MOSS strategy. The MOSS strategy, in comparison to the NSS strategy, possesses the potential to considerably enhance the recognition capabilities of electronic noses. This disparity will be expounded upon both theoretically and experimentally in the following sections.

### 2.2 Computational Fluid Dynamics Analysis of MOSS Strategy

**Figure 2:**
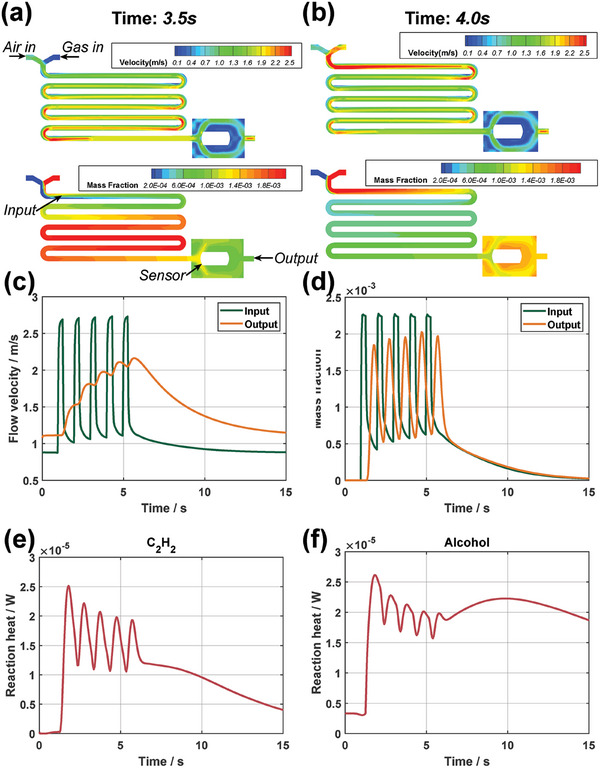
CFD analysis of MOSS strategy. a,b) The velocity field and mass fraction field at two moments (3.5 and 4.0 s) show fast and differentiated dynamics. c,d) The velocity and mass fraction curves at input and output cross‐sections are presented. The same curves are obtained from both experiments of acetylene and ethanol. (e,f) The reaction heat curves of acetylene and ethanol are significantly different, and both are close to the corresponding actual signals in the next two sections.

The computational fluid dynamics (CFD) analysis is conducted to theoretically validate the effectiveness of the MOSS strategy. The gas path of the electronic nose and the bionic lung is simplified into a 2D simulation model (**Figure** **2**a and Figures S3 and S4, Supporting Information). This simulation model comprises a sample sniff inlet, a steady carrier air inlet, a mixing pathway, and a sensor chamber. Inside the sensor chamber, the CTL sensor is strategically positioned as an obstacle, with its windward side designated as the reaction surface where VOCs are oxidized, resulting in luminescence emission. To simulate the signal generated by the sensor luminescence, the CTL reaction is simplified to a VOC oxidation reaction occurring at the surface, and the heat of reaction is employed as the response signal. It is noteworthy that the simulation model simplifies the bionic lung into the sample sniff inlet with a constant input gas composition. The simulated sample sniff from the bionic lung is achieved by adjusting the flow velocity at the sample sniff inlet. When the sample sniff inlet flow rate is set above zero, indicating that the bionic lung is inhaling the sample gas, it travels through the mixing pathway and reaches the reaction surface in the sensor chamber for sensing. The detailed information of the CFD model can be found in Experimental Section.

CFD experiments are conducted using acetylene‐air and ethanol‐air mixtures. For each experiment, the mass fraction of the measured VOC in air is set to 0.03 at the sample sniff inlet. The flow rate at the sample sniff inlet is set to a square wave with a frequency of 1Hz and a duty cycle of 0.3. The flow velocity and sample mass fraction at two pathway cross‐sections, that is, input and output in Figure 2a, are investigated. First, the input cross‐section is quite close to the sniff inlet and thus the gas properties here should be similar to the latter, that is the flow velocity and sample mass fraction at input are both square‐like waves (Figure 2c,d). Then, due to the compressibility of gas and the distance between the output cross‐section and the input cross‐section, the gas property curves at output should have a certain delay compared to those at input. Subsequently, the curve of sample mass fraction at output is delayed sine‐like waves with overlaps. However, the curves of flow velocity at output is a rising sawtooth wave that is obviously different from the mass fraction curve. It can be seen that there is a mismatch between the flow velocity field and the gas composition field during the MOSS process, and this mismatch is evident when comparing the two fields at two instants, that is, 3.5 and 4 s (Figure 2a,b). The intuitive explanation for this phenomenon is that the flow transfer of compressible gases is affected by pressure, while component diffusion is negligible with pressure, so that the mass fraction curve at output is more similar to that at Input compared to the flow rate curve. Besides, the simulation experiments for both gases under test show essentially the same flow velocity and composition fields, illustrating the consistency of gas flow under the MOSS strategy. The video of CFD simulation process can be found in Supporting Information.

Above simulation results demonstrate that the MOSS strategy can provide a sampling process with complex temporal features and good consistency. Therefore, the Arrhenius‐equation‐based oxidation reaction models emerge as pivotal in determining distinct reaction heat profiles for various sample species. The reaction heat curves of acetylene and ethanol have obviously different waveforms (Figure 2e,f). Both curves consist of four consecutive peaks and an independent tail peak. The formation of consecutive peaks is due to the rising accumulation of flow velocity which accelerates the response and recover of sensors. Then after the last sniff in MOSS, the flow velocity begins to slow down and the last signal peak will consequently have longer response and recover times than the previous peaks. Comparing the results of two experiments, the consecutive peaks of acetylene have better resolution than that of ethanol, while the tail peak of acetylene is narrower and lower than that of ethanol. According to the Arrhenius‐equation‐based models, the difference between the two reaction heat curves can be explained as being mainly influenced by the different reaction exponents of the measured gases. The reaction exponents for acetylene and ethanol are 0.5 and 0.15, so the same mass fraction curve results in different reaction heat curves, the former being more similar to the mass fraction curve and the latter being flatter with a more pronounced and prolonged tail peak. This disparity in reaction heat curves demonstrates that one CTL sensor can readily distinguish between acetylene and ethanol with the aid of the MOSS strategy. Subsequent experiments will further substantiate the enhanced recognition capability facilitated by the MOSS strategy.

### 2.3 MOSS‐Based VOC Recognition Experiments

**Figure 3:**
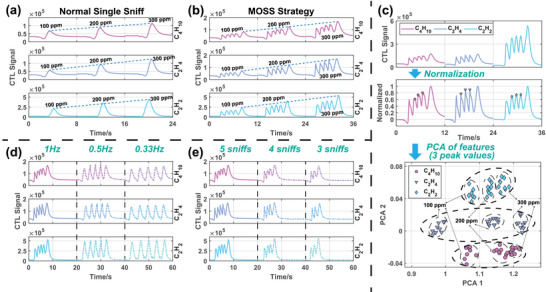
VOC recognition experiments. a) With traditional “normal single sniff” (NSS) strategy, one single CTL sensor is able to recognize the concentrations of VOCs, but not their species. b) With MOSS strategy, the same CTL sensor can response not only linearly to VOC concentrations, but also differently to VOC species. c) The MOSS signals of three species with three concentrations of VOC gases are normalized, and the middle three peaks are taken as features and subjected to PCA. The species of C_4_H_10_, C_2_H_4_, and C_2_H_2_ are successfully recognized. Besides, the sub‐clusters reflect the concentration of the measured VOCs are marked. d) The CTL signals of C_2_H_4_ and C_2_H_2_ at 300ppm are measured utilizing the MOSS strategy with different sniffing frequencies. e) The CTL signals of C_2_H_4_ and C_2_H_2_ at 300ppm are measured utilizing the MOSS strategy with different sniffing numbers and a sniffing frequency of 1Hz.

The VOC recognition via a single gas sensor is often regarded as a challenging task. Previous researchers delved into sensing mechanisms of various gases in search of distinctive signal features.^[^ ^46^, ^47^, ^48^ ^]^ This section demonstrates how adopting the multiple overlapping sniffs (MOSS) strategy provides more differentiated sensing signals, effectively enhancing VOC recognition. In this experiment, only sensor No.1 is applied to recognize three kinds of standard VOC gases, that is, butane, ethylene and acetylene (C_4_H_10_, C_2_H_4_, and C_2_H_2_). It can be seen that, with the normal single sniff (NSS), the CTL sensor can response to VOC gases with a single peak whose magnitude is linearly proportional to the VOC concentration (**Figure** **3**a and Figure S5, Supporting Information). However, NSS strategy yields response signals consisting of analogous single peaks across diverse gases, consequently lacking the crucial discriminatory details needed for differentiation. To address this, the MOSS strategy is adopted, involving five consecutive sniffs at a frequency of 1Hz for gas sampling. The response signals generated by the identical CTL sensor distinctly exhibit dissimilar waveforms in the presence of various gases (Figure 3b and Figures S6 and S7, Supporting Information). Specifically, the relative magnitudes of five signal peaks correlate with the VOC species. Besides, the maximum magnitudes, represented by the last peaks, maintain a linear relationship with the VOC concentrations (Figure S8, Supporting Information). Since the absolute magnitudes of CTL signals are determined by both VOC concentrations and VOC species, by normalizing the signal magnitudes, the normalized signals are presented to recognize VOC species based on the peak relative magnitudes (Figure 3c). Subsequently, the middle three peak values within the normalized signals are selected as feature values. These selected values then undergo principal component analysis (PCA) to reduce the dimensionality and showcase the recognition performance for 78 groups of VOC signals. The detailed data processing procedure can be seen in Experimental Section. It should be highlighted that although three concentrations of each gas, that is, 100, 200, 300ppm, are included, PCA still shows excellent recognition performance of different VOC gases. In summary, the MOSS strategy enables one single CTL sensor to rapidly recognize both the species and concentrations of VOCs.

In order to further discuss the design of MOSS strategy, experiments of different sniff frequency and number are also carried out. As the sniff frequency reduces, the five peaks are corresponding further apart (Figure 3d). At the same time, the amplitudes and waveforms of signal peaks are more consistent, and there is less overlap between peaks and less narrowing of middle peaks. In fact, according to the CFD analysis above, the reduced sniff frequency can lead to less sample accumulation and weaker interaction between sniffs, resulting in signal peaks that are more independent, that is, more like the peaks in the NSS strategy. Moreover, as the number of sniffs decreases, the number of signal peaks also decreases accordingly (Figure 3e). Nevertheless, due to the constant sniff frequency, the signal peak pattern does not change significant, that is, there are still multiple consecutive peaks and one tail peak. Besides, only the number of continuous peaks is decreasing with the number of sniffing, which is also consistent with the explanation in CFD analysis. Hence, it can be concluded that the key to designing MOSS strategy is the sniff frequency which ensures that there is both discrimination and overlap between multiple sniffs.

### 2.4 MOSS‐Based Liquor Recognition Experiments

**Figure 4:**
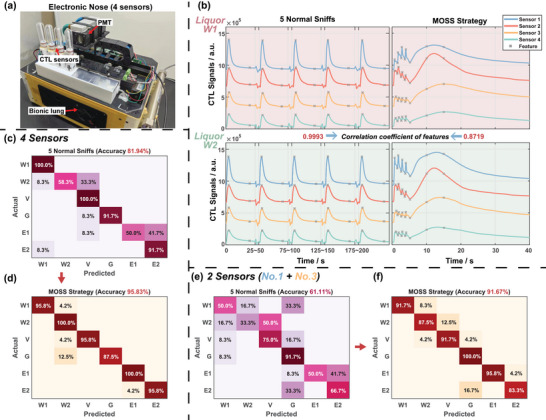
Liquor recognition experiments. a) Diagram of experiment equipment. b) Compared to five normal sniffs (NSS) strategy, MOSS strategy results in more remarkable signal differences between two whiskys (W1 and E2). The selected feature values are marked in this figure. The last 25 s of each signal segment under the NSS strategy are omitted because the signal returns to baseline after 25 s. The decrease in correlation coefficients of features from NSS to MOSS strategy indicates an increase in the divergence of signals. c,d) Confusion matrices for NSS and MOSS strategies, with the latter clearly having better accuracy. e,f) Confusion matrices of two‐sensor experiments. Only the signals of sensors No.1 and No.3 are applied in the recognition experiments. The recognition accuracy of NSS strategy is significantly reduced, while that of MOSS strategy still exceeds 90%.

Liquor recognition stands as a prominent application scenario for electronic noses. However, compared to ethanol, the primary constituent of liquors, the number of aroma compounds significantly influencing liquor odors is relatively limited. Thus, the augmentation of gas sensors in electronic noses is often necessary to discern aroma compounds masked by ethanol. In this experiment, a 4‐CTL‐sensor electronic nose (**Figure** **4**a) is employed for the recognition of six liquors (**Table** **1**), with their odors collected via headspace sampling. The aromatic hydrocarbon contents of the 6 liquors are analyzed by gas chromatography‐mass spectrometry, revealing differences, albeit relatively trace (Figures S9– S11, Supporting Information). The CTL signals for two whiskeys under the NSS and MOSS strategies are displayed (Figure 4b). For the NSS strategy, five sequential sniffs are shown, each with a 50‐s interval, while the MOSS strategy involves five consecutive sniffs with a sniffing frequency of 1Hz. Under the traditional NSS strategy, the response signals for different liquors show little noticeable difference, while the MOSS strategy yields discernible distinctions. Quantitatively, the correlation coefficients between signal feature vectors of two whiskeys under the NSS and MOSS strategies are 0.9993 and 0.8719 respectively (Figures S12– S14, Supporting Information). This indicates that, without the addition of any sensors, the MOSS strategy enriches signal information, amplifying distinctions in liquor signals.

**Table 1:** The list of measured liquors.

Abbreviation	Name	Alcohol content
**W1**	Chivas Regal Aged 12 Years Blended Scotch Whisky	40%
**W2**	Ballantine's Finest Blended Scotch Whisky	40%
**V**	Absolut Vodka	40%
**G**	Bombay Sapphire London Dry Gin	47%
**E1**	Niulanshan Erguotou	46%
**E2**	Ren Star Erguotou	56%

A typical machine learning algorithm, namely support vector machine (SVM), is utilized to recognize liquor signals. The SVM input vector encompasses 40 features derived from electronic nose signals. For the MOSS strategy, features are manually selected from four consecutive peaks, including the peaks, troughs, and two points at fixed times (10 and 15 s) to capture the magnitude and waveform of the tail peak, resulting in ten features per sensor. For the fair comparison of NSS with MOSS strategy, five sequential normal single sniffs (NSSs) are taken as one set of signals and compared with signals from the MOSS strategy. For the NSS strategy, the features are also selected from peaks and troughs, amounting to ten features per sensor (Figure 4b). 72 groups and 150 groups of test signals are collected Using NSS and MOSS strategies. Fivefold cross validations (Figure S15, Supporting Information) are conducted to assess the recognition performance of both strategies. Fivefold cross validations divide the signal dataset into five subsets, with each subset being predicted by an SVM trained on the other four subsets. The prediction accuracy, representing the recognition performance, is depicted by confusion matrices. Diagonal elements signify the prediction accuracy of each liquor, while off‐diagonal elements indicate the proportion of false predictions. From NSS to MOSS, the prediction accuracy rises from 81.94% to 95.83%, proving that MOSS can greatly enhance the liquor recognition capability of electronic noses (Figure 4c,d).

Furthermore, to verify that the MOSS strategy can overcome the equipment limitation of electronic noses, another experiment is carried out with even fewer sensors to recognize six kinds of liquors. Specifically, it is assumed that the CTL electronic nose consists of only two sensors: sensors No.1 and No.3 (Figure 4e). In other words, from the above‐mentioned dataset of six liquors, only the test signals from sensor No.1 and No.3 are used. The selection of sensors No.1 and No.3 is based on the representation of two typical CTL sensing materials, namely Al_2_O_3_ and SiO_2_. Due to the difference in microstructure, these two materials are chosen to best represent the electronic nose sensor array manufactured in reality, wherein different materials are employed to diversify the cross‐response to odors. Consequently, the input vector for SVM is a 20D vector for both NSS and MOSS strategies. Utilizing the same fivefold cross validations, the prediction accuracy of NSS and MOSS strategies is compared. As the sensor count decreases, the prediction accuracy of NSS notably drops to 61.11% (Figure 4e), indicating a significant impact of sensor number on the recognition capability of electronic noses. However, with regard to the MOSS strategy, the prediction accuracy only decreases to 91.67%, which remains within an acceptable range for recognition tasks (Figure 4f). Therefore, the use of the MOSS strategy in electronic noses demonstrates remarkable robustness even in scenarios with limited sensors, which is particularly valuable in the present context where electronic nose sensor arrays remain relatively small.

## 3 Conclusion

Sniffing, as a fundamental behavior of terrestrial vertebrate olfaction, has been introduced to electronic noses in the form of the MOSS strategy and significantly enhanced the recognition performance of various VOCs and liquor odors. The MOSS strategy, akin to a rat eagerly seeking cheese, enables the electronic nose to artificially control the inhalation of odorants, generating dynamic airflows that enhance signal features. The CFD simulations demonstrate that MOSS can repeatedly induce periodic variations in the odorant field, effectively utilizing sensor transient responses. Moreover, it is proved that the difference in response kinetics of VOC gases directly leads to the difference in sensor signals. Hence, this strategy holds theoretical applicability to a broader array of gas sensors than just CTL sensors, given the pervasive nature of differences in response kinetics to various gas molecules within the same sensor. This paper, employing the MOSS strategy, demonstrates that a single CTL sensor can recognize VOC components by simply extracting peak features, bypassing the cumbersome pre‐separation process and complex machine learning algorithms in previous single‐sensor detection studies. The liquor recognition experiments also vividly showcase the recognition enhancement of the MOSS strategy on electronic noses. Specifically, the 4‐CTL‐sensor array using the conventional NSS strategy attains a recognition accuracy of only 81.94% for six liquors, whereas when employing the MOSS strategy, the same sensor array achieves an accuracy of 95.83%. Remarkably, even with just two CTL sensors, a recognition accuracy of 91.67% for the six liquors is accomplished using the MOSS strategy.

This study emphasizes the importance of gas flow modulation for enhancing gas sensor performance. Bionic sniffing strategies, which enable repeated flow switching akin to cyclic breathing and concentration of odorants on sensors through overlapping sniffs, hold promise for future research in electronic noses to improve the speed and accuracy of odor recognition. The proposed MOSS strategy has demonstrated its effectiveness in enhancing CTL electronic nose recognition, and its applicability to other electronic noses will be further explored in upcoming studies. Additionally, this strategy is anticipated to find applications in various automated odor recognition scenarios, including food quality inspection, clinical diagnosis, explosive detection, and more.

## 4 Experimental Section

### Experiment Equipment and Materials

In this paper, an electronic nose with a bionic lung is designed as shown in Figure 4a. A injection pump, bought from Longer Precision Pump Co., Ltd., served as the bionic lung with a total injection volume of 25mL and an maximum injection speed of 1slm. To implement the MOSS strategy, the injection process of the injection pump can be controlled through programs. Meanwhile, the sensor array of the electronic nose contained four cataluminescence (CTL) sensors. Inside the transparent quartz tube of each sensor, the catalysts are attached to a temperature‐controlled heated ceramic rod, and the measured VOC gas is oxidized and emit weak luminescence as it passed over the surface of catalysts. The luminescence of CTL reaction was collected and converted into digital signals by a photomultiplier tube (PMT), purchased from Hamamatsu Photonics Co., Ltd. It is worth mentioning that the previous study^[^ ^46^ ^]^ has proved that the air flow inside the CTL sensor is laminar and can be simplified into a 2D model. The CTL sensor chamber is composed of a ϕ20 × 50 mm quartz tube which is represented in the simulation model as a 40 × 30 mm rectangle. As the four sensors are independent, in the experimental setup, the gas path between the injection pump and the sensors included additional equipment such as the plasma‐assisted unit and multi‐channel valves. The length of the tubing between them was ≈800 mm, with an average diameter of ϕ3.5 mm. Consequently, a pipe of ≈800 mm in length and 4 mm in width is designed in front of the sensor chamber in the simulation model (Figure S4, Supporting Information). During the experiments, the set carrier gas flow rate is 0.5 slm, with a maximum injection flow rate of 1.0slm. In the simulation model, the flow rates at the two outlets were set to 0.9 and 2.0 m s^−1^, respectively. In summary, the simulation model ensured similar airflow conditions to those in the experimental equipment, thus enabling relatively consistent simulation results. Meanwhile, the measured gas molecules can reach the sensor surface within 2 s of injection. According to the VOC recognition experiments, it took ≈6 s of continuous carrier air to completely eliminate the signal of 5mL VOC gas, that is, ≈50mL of carrier air is required.

The catalysts of four CTL sensors were Al_2_O_3_, Al_2_O_3_/Ni^2 +^, SiO_2_/Cu^2 +^ and SiO_2_/Mn^2 +^, respectively. Nanopowder of Al_2_O_3_ and SiO_2_ are used as the catalyst substrates and solutions of NiSO_4_, CuSO_4_, and MnCl_2_ are used for ion doping. A sol–gel method^[^ ^49^ ^]^ was employed to synthesize the catalysts. Briefly, 0.5g of nanopowder was dissolved in 50mL of 0.2mol ion aqueous solution and stirred at room temperature for 6h. Next, the solution was centrifuged to obtain the precipitated nanopowder. After washing with deionized water, the nanopowder was drained at 80°C for 5h and then calcinated in the muffle furnace at 600 °C for 12h to activate its catalytic properties. Last, the active catalysts were attached on the ceramic rods and heated to 280 °C for detection. Butane, ethylene, and acetylene gases were balanced with nitrogen gas and provided by Beijing Haipu‐Gas Co., Ltd. All above‐mentioned reagents were at least of analytical‐reagent grade. Besides, liquors in the experiments are all commercially available products.

### Experiment Setups

In the VOC recognition experiments, butane, ethylene, and acetylene gas samples were collected in sampling bags at three concentrations (100, 200, 300ppm) for a total of nine samples. For both NSS and MOSS strategies, the same parameters were taken for each sniff, namely, a sniff volume of 5mL and a sniff duration of 0.3s. Only one sniff was performed in the NSS strategy, while five sniffs with 1Hz frequency were performed in the MOSS strategy. In the case of the liquor recognition experiment, where both strategies necessitate five injections, the injection pump initially inhaled in 25mL of the measured gas. Subsequently, 5mL of gas was injected sequentially at specified time intervals (50 and 1s). 20 mL of each liquor in **Table** **1** was placed in a 50 mL headspace sample bottle and left to stand for 20 min before dynamic headspace sampling. Besides, the sniff parameters in liquor recognition experiments were the same as described above.

In the VOC identification experiments, each of the 9 VOC gases was measured ten times using both NSS and MOSS strategies, showing a high level of consistency in the testing signals. During PCA for VOC recognition, data points with maximum values exceeding 3σ were excluded. Consequently, 78 groups of signals were involved in the recognition of VOC species. While in the liquor identification experiments, each of six liquors was tested 12 times using the NSS strategy, and 24 times using the MOSS strategy. Consequently, there were 72 groups and 144 groups of signals, respectively, participating in the recognition experiment (Figures S5– S7, S12, and S13, Supporting Information).

### Nature of Electronic Nose Data

Using a time series signal *X*_*ij*_(*t*) to express the response value of a certain sensor *i* to an odor *j* at the *t* moment. When a CTL sensor was in operation, the PMT continuously captured its luminescence with a sampling time of 0.1s and converted into the signal *X*_*ij*_(*t*). In the absence of the measured VOC gas, the signal *X*_*ij*_(*t*) stabilized near the baseline. When the measured VOC gas was introduced, the time it took for the signal *X*_*ij*_(*t*) to reach its maximum value max{*X*_*ij*_(*t*)} was defined as the response time. Besides, the time it took to return to the baseline from the maximum value max{*X*_*ij*_(*t*)} was referred to as the recovery time. For the conventional NSS strategy, CTL sensors consistently demonstrated response times of less than 5 s (Figures 1, 3, and 4b). However, for the MOSS strategy, where the introduction of gas occured in multiple stages, the standard definition of response time may not be directly applicable. As this had little impact on the conclusions of this study, a detailed definition had not been pursued yet.

The preprocessing of CTL signals primarily aimed to remove the sensor's luminescent background noise, referred to as the signal baseline. In this study, the mean of the signals in 1s before gas introduction was used to represent the signal baseline. Therefore, the preprocessing algorithm for transforming the raw signal $X_{ij}^{r}\left(t\right)$ into the signal *X*_*ij*_(*t*) can be expressed as

(1) $$\begin{array}{c} X_{ij}\left(t\right)=X_{ij}^{r}\left(t\right)-\frac{1}{10}\sum_{k=-10}^{-1}X_{ij}^{r}\left(k\right) \end{array}$$

If there are *m* odors were measured by *n* sensors, the response of the *n*‐sensor array to an odor *j* can be described into a matrix:

(2) $$\begin{array}{c} X_{j}=\left(\begin{array}{cccc} X_{1j}\left(0\right) & X_{1j}\left(1\right) & \cdots & X_{1j}\left(T\right)\\ X_{2j}\left(0\right) & X_{2j}\left(1\right) & \cdots & X_{2j}\left(T\right)\\ \vdots & \vdots & X_{ij}\left(t\right) & \vdots \\ X_{nj}\left(0\right) & X_{nj}\left(1\right) & \cdots & X_{nj}\left(T\right) \end{array}\right) \end{array}$$

In this paper, multiple measurements were performed for each type of measured gas. Due to potential environmental interference, some measurements may exhibit significant errors. To address this, the maximum signal max{*X*_*ij*_(*t*)} was employed as a metric within the dataset for a given gas type. The signal *X*_*j*_ whose maximum signal max{*X*_*ij*_(*t*)} exceeded the range of *E*(max{*X*_*ij*_(*t*)}) ± 3σ will be eliminated. The detailed signal data can be seen in Supporting Information.

### PCA Process of VOC Recognition Experiments

In the VOC recognition experiments, the principal component analysis (PCA) was adopted for the recognition of VOC species. PCA was a common pattern recognition algorithm used to analyze data obtained from electronic noses.^[^ ^2^ ^]^ For the MOSS strategy, the three middle peaks in the signal were utilized as features. To mitigate the influence of signal amplitude on identification, a normalization process was applied to the measured signal *X*_1*j*_(*t*) to obtain the normalized signal $X_{1j}^{n}\left(t\right)$

(3) $$\begin{array}{c} X_{1j}^{n}\left(t\right)=\frac{X_{1j}\left(t\right)-X_{1j}\left(0\right)}{\max\left\{X_{1j}\left(t\right)\right\}-X_{1j}\left(0\right)} \end{array}$$

The peak features of $X_{1j}^{n}\left(t\right)$ are manually selected as (*f*_1_, *f*_2_, *f*_3_). Then, MATLAB's built‐in PCA function was employed to reduce the dimensionality of the features (*f*_1_, *f*_2_, *f*_3_) to a 2D space composed of principal components (PCA1, PCA2). PCA1 and PCA2 represented the most significant patterns in the dataset. PCA1 captured the largest source of variability, while PCA2, orthogonal to PCA1, captured the second largest source of variability. Together, they provide a condensed representation of the data, allowing for visualization and analysis of the essential features that distinguish different VOC species (Figure 3c).

### CFD Functions

The CFD analysis was performed with Ansys Fluent. The transient explicit density‐based solver in Ansys Fluent was employed in the investigation of compressible flow through the 2D model in Figure 2a. Shaer stress transport model *k* − ω turbulent model was applied to solve the Reynolds average Navier Stokes equations. Moreover, the species transport equations and the laminar finite‐rate model were introduced to simulate the diffusion and oxidation of the measured gas. It should be noted that in this study, the CFD simulations were specifically conducted for the CTL reactions of acetylene and ethanol. Given that the catalytic oxidation reactions of these two compounds involve complete irreversible oxidation, Arrhenius‐based models were employed. The simulation model was designed to illustrate that the differences in response mechanisms for different gases can be manifested as waveform disparities in the time‐series signals under the MOSS strategy. Hence, if future studies involved different chemical reaction models for investigating other analytes, it was anticipated that the MOSS strategy will still yield waveforms in distinct forms. Additionally, since the pressure on the sensor surface remained basically constant and the gas concentration was low, the instantaneous adsorption of gas molecules on the sensor surface can be assumed to be approximately proportional to the gas concentration.^[^ ^50^ ^]^ Consequently, the simulation at this stage overlooks the adsorption/desorption equations.

The governing equations are listed as following. First, the governing equations for conservation of mass, momentum, and energy equations are expressed as

(4) $$\begin{array}{c} \frac{\partial \rho }{\partial t}+\nabla \cdot \left(\rho \vec{v}\right)=0\\ \frac{\partial }{\partial t}\left(\rho \vec{v}\right)+\nabla \cdot \left(\rho \vec{v}\vec{v}\right)=-\nabla p\\ \frac{\partial }{\partial t}\left(\rho E\right)+\nabla \cdot \left(\bar{v}\left(\rho E+p\right)\right)=-\nabla \cdot \left(\sum_{i}h_{i}{\vec{J}}_{i}\right) \end{array}$$

where ρ, $\vec{v}$, *p*, and *E* are the density, velocity, pressure, and energy of the gaseous mixture. Besides, *h*_*i*_ and ${\vec{J}}_{i}$ are the enthalpy and diffusion flux of species *i*, making up the enthalpy transport term. In order to form closed equations, ideal gas law should be included as

(5) $$\begin{array}{c} \rho RT=pM_{W} \end{array}$$

where *T* and *M*_W_ are the temperature and molecular weight of the mixture. *R* is the universal gas constant. Moreover, the enthalpy transport term due to species diffusion in the energy conservation equation should be calculated, and the species diffusion also affected the physics properties of the mixture, such as ρ and *M*_W_. Hence, the species transport equations are introduced as

(6) $$\begin{array}{c} \frac{\partial }{\partial t}\left(\rho Y_{i}\right)+\nabla \cdot \left(\rho \vec{v}Y_{i}\right)=-\nabla \cdot {\vec{J}}_{i}+R_{i}\\ {\vec{J}}_{i}=-\rho D_{i,m}\nabla Y_{i} \end{array}$$

where *R*_*i*_ is the production rate of species *i* by chemical reaction. It should be mentioned that the second equation of Equation (6) is the classic Fick's Law, and *D*_*i*, *m*_ is the mass diffusion coefficient for species *i* in the mixture.

Last, the laminar finite‐rate model computes the chemical source terms using Arrhenius expressions

(7) $$\begin{array}{c} R_{i}=\left(v_{i}^{\prime \prime }-v_{i}^{\prime }\right)\left(k_{f}\prod_{j}^{N}{\left[C_{j}\right]}^{\left(\eta_{j}^{\prime }+\eta_{j}^{\prime \prime }\right)}\right) \end{array}$$

where $v_{j}^{\prime }$, $\eta_{j}^{\prime }$ are the stoichiometric coefficient and the rate exponent for reactant species *j*, $v_{j}^{\prime \prime }$, $\eta_{j}^{\prime \prime }$ are the stoichiometric coefficient and the rate exponent for product species *j*, *C*_*j*_ is the molar concentration of species *j*, and *k*_f_ is the forward rate constant for the reaction.

In brief, Equations ((4), (5), (6), (7)) form the closed governing equations to simulate the MOSS process.

## Conflict of Interest

The authors declare no conflict of interest.

## Supporting information

Supporting Information

Supplemental Movie 1

## Data Availability

The data that support the findings of this study are available from the corresponding author upon reasonable request.
