# Supplementary material for: Sniffing Like a Wine Taster: Multiple Overlapping Sniffs (MOSS) Strategy Enhances Electronic Nose Odor Recognition Capability
Source: Adv Sci (Weinh). 2023 Dec 14;11(7):2305639. doi: 10.1002/advs.202305639 (PMC10870059; doi:10.1002/advs.202305639)
Supplement: Supplementary file 1 — Supporting Information [file ADVS-11-2305639-s002.pdf]

ADVANCED SCIENCE
Open Access

Supporting Information

for *Adv. Sci.*, DOI 10.1002/advs.202305639

Sniffing Like a Wine Taster: Multiple Overlapping Sniffs (MOSS) Strategy Enhances Electronic Nose Odor Recognition Capability

*Luzheng Liu, Na Na, Jichuan Yu, Wenxiang Zhao, Ze Wang, Yu Zhu and Chuxiong Hu**

Supporting Information

Sniffing like a Wine Taster: Multiple Overlapping Sniffs (MOSS) Strategy Enhances Electronic Nose Odor Recognition Capability

*Luzheng Liu, Na Na, Jichuan Yu, Wenxiang Zhao, Ze Wang, Yu Zhu, Chuxiong Hu**

Email: cxhu@tsinghua.edu.cn

This PDF file includes:

Supplementary Text
Figs. S1 to S15
Movies S1

Other Supplementary Materials for this manuscript include the following:

Movies S1

Supplementary Text

Experiment equipment.

The experiment equipment of the cataluminescence (CTL) electronic nose is discussed (Fig.S3). CTL sensing requires a stable physical and chemical environment, so several controllable devices are applied.

First, a sample injection gas path and an air carrier with steady flow are needed. The sample injection is carried out by a injection pump as in Fig.S3. The air carrier is produced by an air pump, and a digital mass flow controller (MFC) is engaged for air flow control. This MFC can ensure the flow error within ±5 standard cubic centimeter per minute (sccm). Then the sample gas and steady air carrier are blended together by a 3-way valve, and flow through a plasma-assisted unit which activates VOCs and oxygen to enhance CTL intensity. Moreover, the gas pathways of the four CTL sensors are connected in parallel, with their respective inlets unified through a 4-channel switching valve. During measurements, only one channel is actively connected at a time. This design ensures the consistency of the gas pathways across all four channels, enabling cross-responses to sample gases.

Next, CTL reaction takes place inside the CTL sensor cavity which is a transparent quartz tube. Inside the cavity, there is a quartz pipe and a ceramic heating rod with catalyst. Activated sample gas with carrier flows through the quartz pipe, from the top of the cavity tube to the bottom of the cavity tube. As gas flows around the ceramic heating rod, heating core inside the rod is continuously heated to a preset temperature. The heating core is controlled by a temperature control board which provides stable temperature within ±1℃. This heating rod ensures that activated sample gas is oxidized on the catalyst surface, emitting a trace amount of light. Furthermore, the light emitted is recorded by a photomultiplier tube (PMT) which contributes the sensing signal. In brief, PMT detects the photon number during each sampling period, and outputs the photon number as a sensing signal. Besides, to avoid additional light background noise, CTL sensors and PMT shall be sealed in a dark chamber.

All the above-mentioned digital devices communicate with a microcontroller through serial ports. To sum up, the experiment equipment can realize automatic CTL test with stable reaction parameters. And the detailed experiment parameters are shown in Table S1.

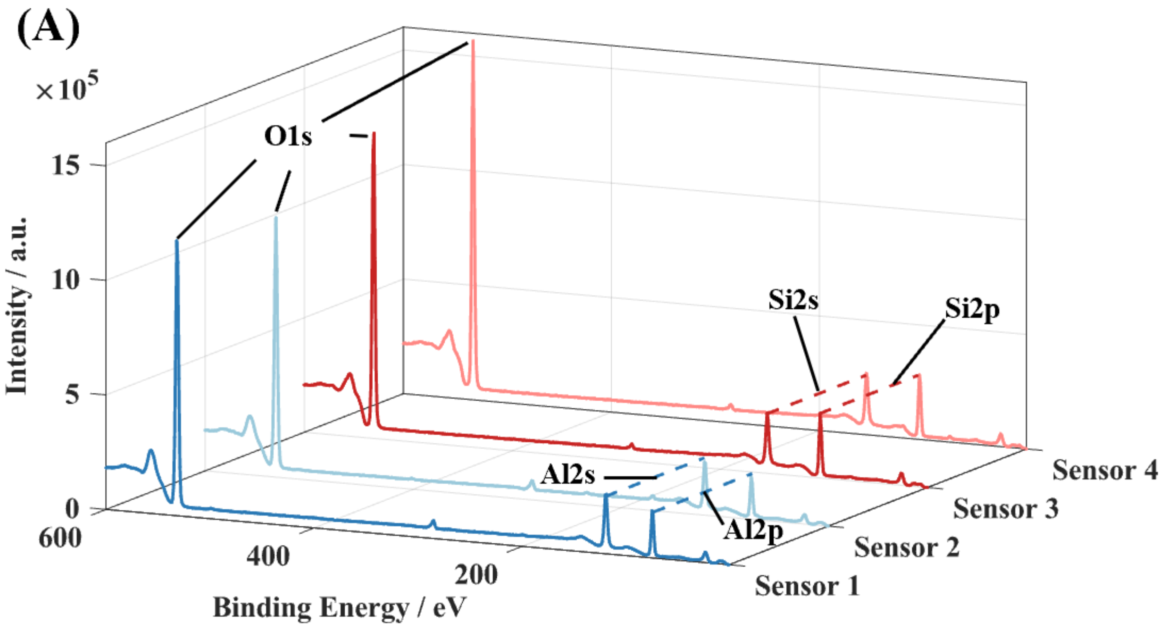

(B)

Sensor No.	Catalyst	Ion wt.%
1	Al_2O_3	-
2	Al_2O_3/Ni^{2+}	1.46
3	SiO_2/Cu^{2+}	1.32
4	SiO_2/Mn^{2+}	3.21

Fig. S1.

The elemental characterization of 4 catalysts. (A) The XPS spectra of 4 catalysts demonstrate that their main composition is the nano metal oxide powder as substrates. (B) The mass percentages of dopant ions in 4 catalysts are measured by ICP-OES. Despite the low doping ion content, different catalysts are able to produce differentiated responses to VOC gases.

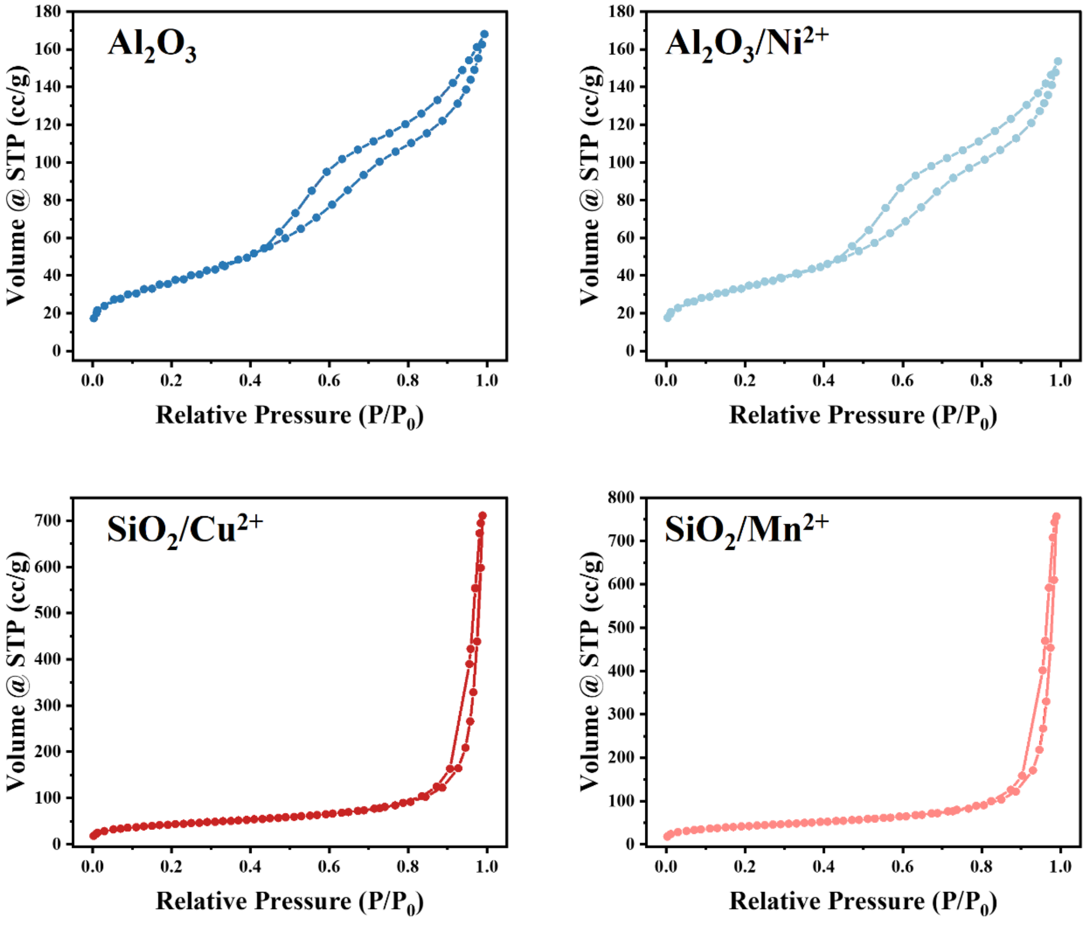

Fig. S2.

The BET characterization on 4 catalysts. It can be seen that catalysts with same substrates have similar adsorption properties, and all 4 catalysts are favorable to adsorb gas molecules and produce the CTL reaction.

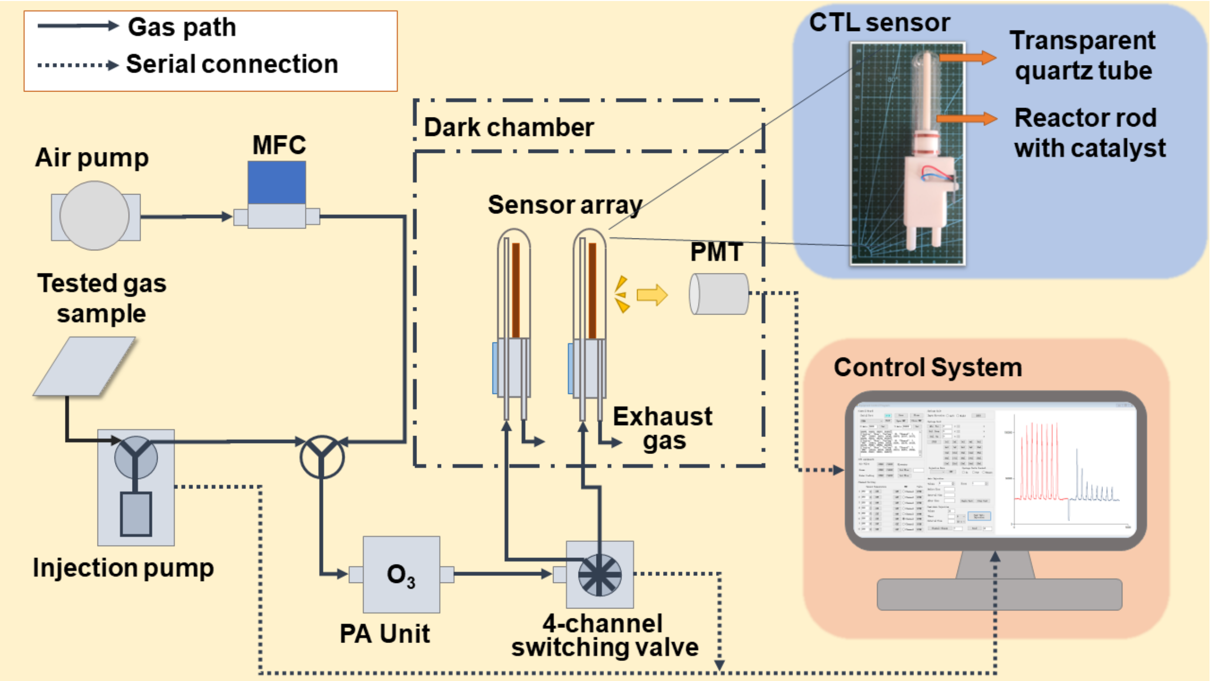

Fig. S3.

The Experiment equipment of the cataluminescence (CTL) electronic nose. The equipment features a parallel unidirectional gas pathway, where the measured gas flows through various components of the setup from the injection pump, and eventually exits from the sensor outlet. Each component of the equipment can be controlled via the upper computer to ensure the stability of the sensor's reaction conditions.

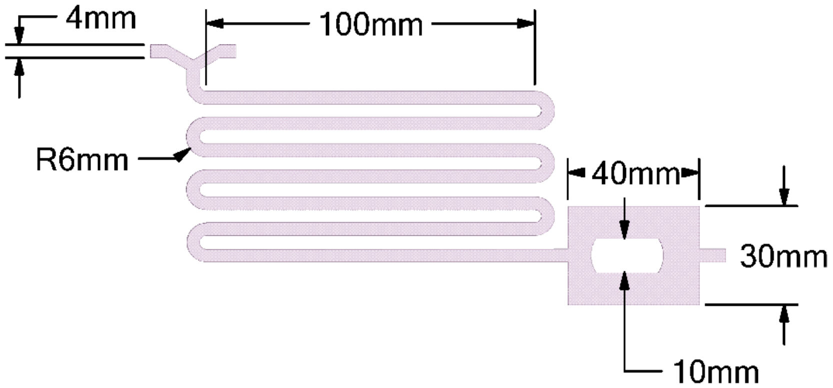

Fig. S4.

The two-dimensional model for CFD simulation. The simulation model utilizes a three-way valve to simulate the sample injection module of the experimental setup. It employs a tubing of approximately 800 millimeters in length to replicate the equipment's tubing. Additionally, a rectangular chamber is employed to simulate the chamber of the CTL sensor.

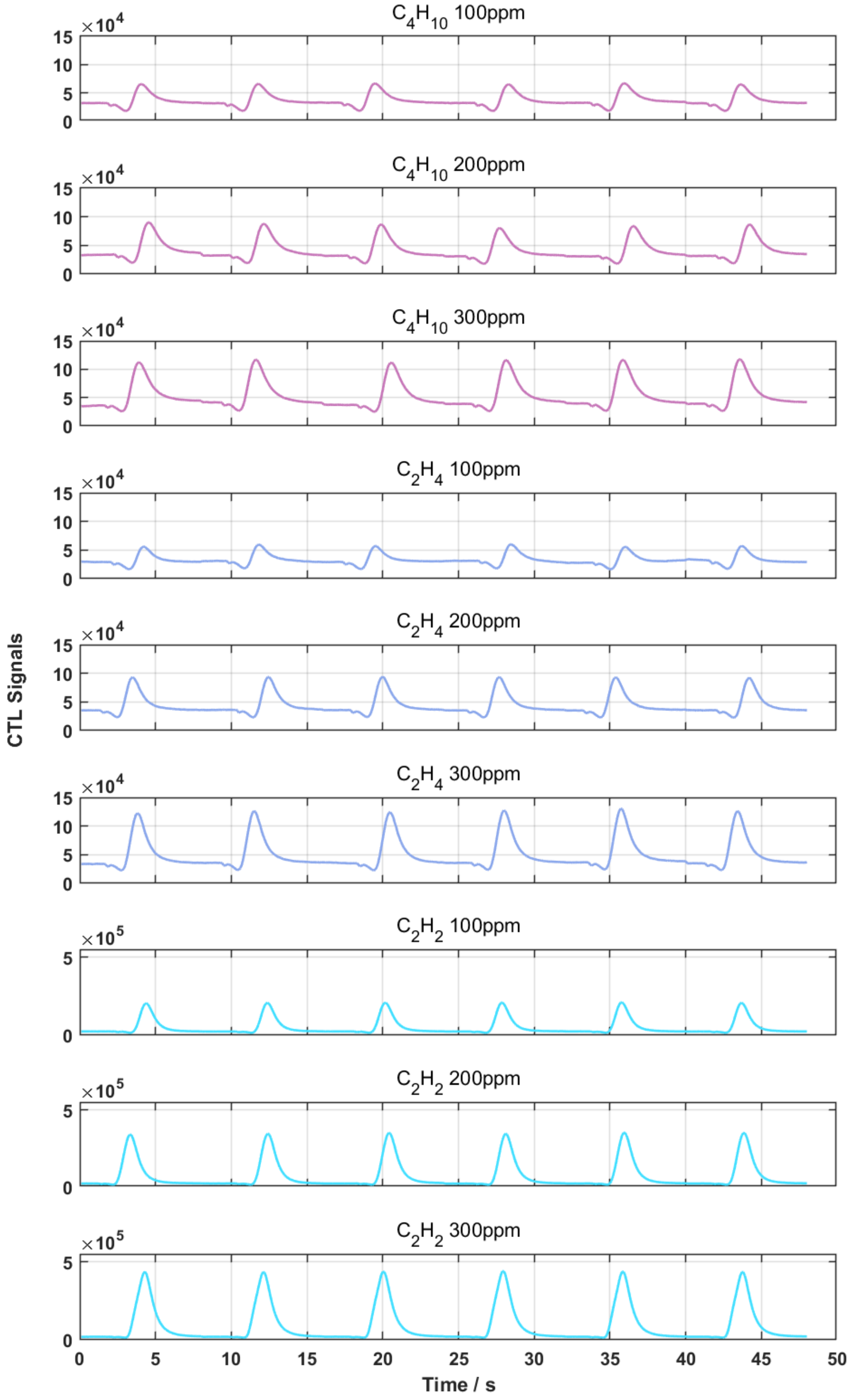

Fig. S5.

Signal examples of 9 VOC samples measured with the NSS strategy. It can be observed that there is good consistency in the testing signals for each gas sample. The testing for each gas was repeated 10 times. In this figure, to showcase the details of the signal waveform, 6 groups of signals are displayed.

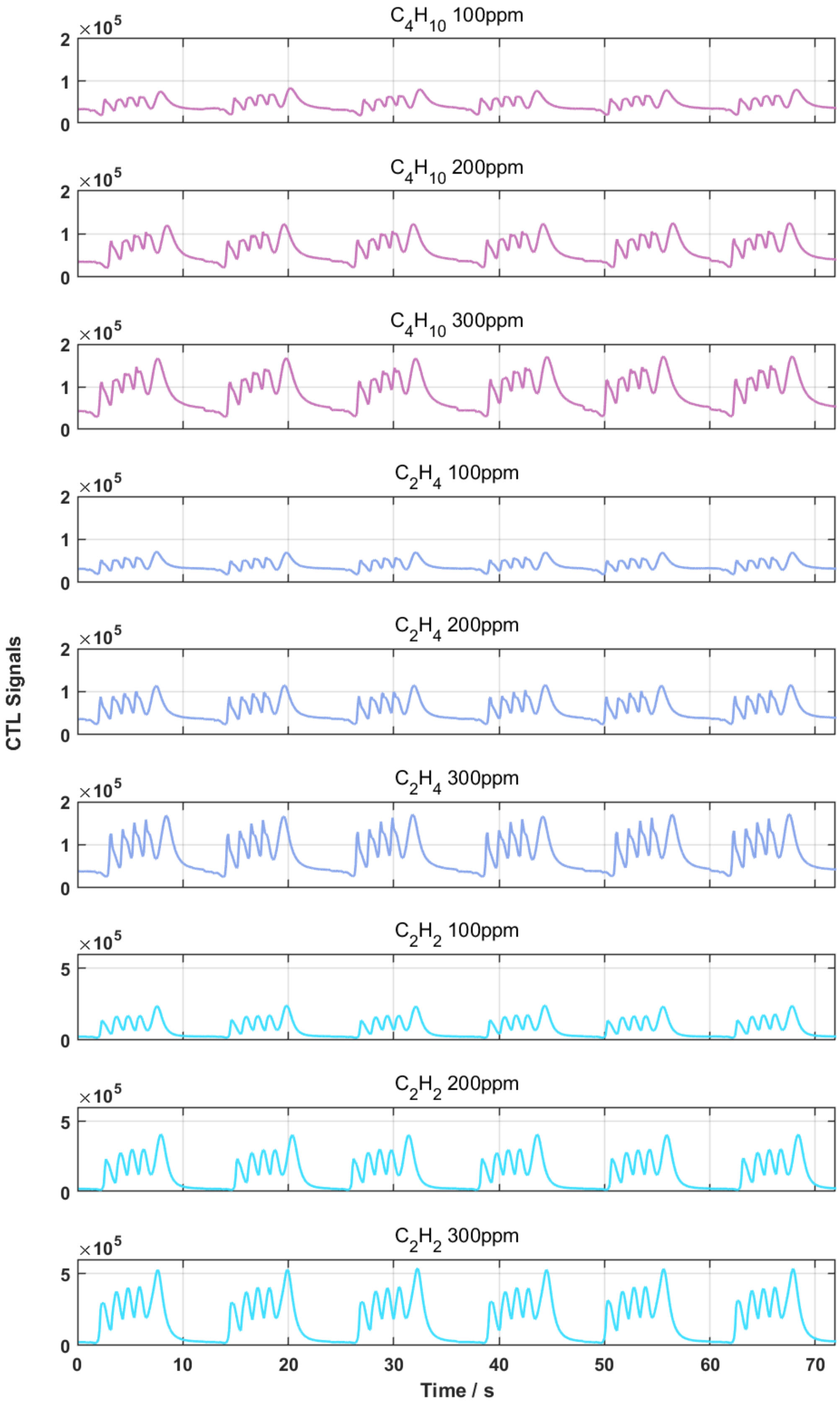

Fig. S6.

Signal examples of 9 VOC samples measured with the MOSS strategy. It can be observed that there is good consistency in the testing signals for each gas sample. The testing for each gas was repeated 10 times. 6 groups of signals are displayed in this figure.

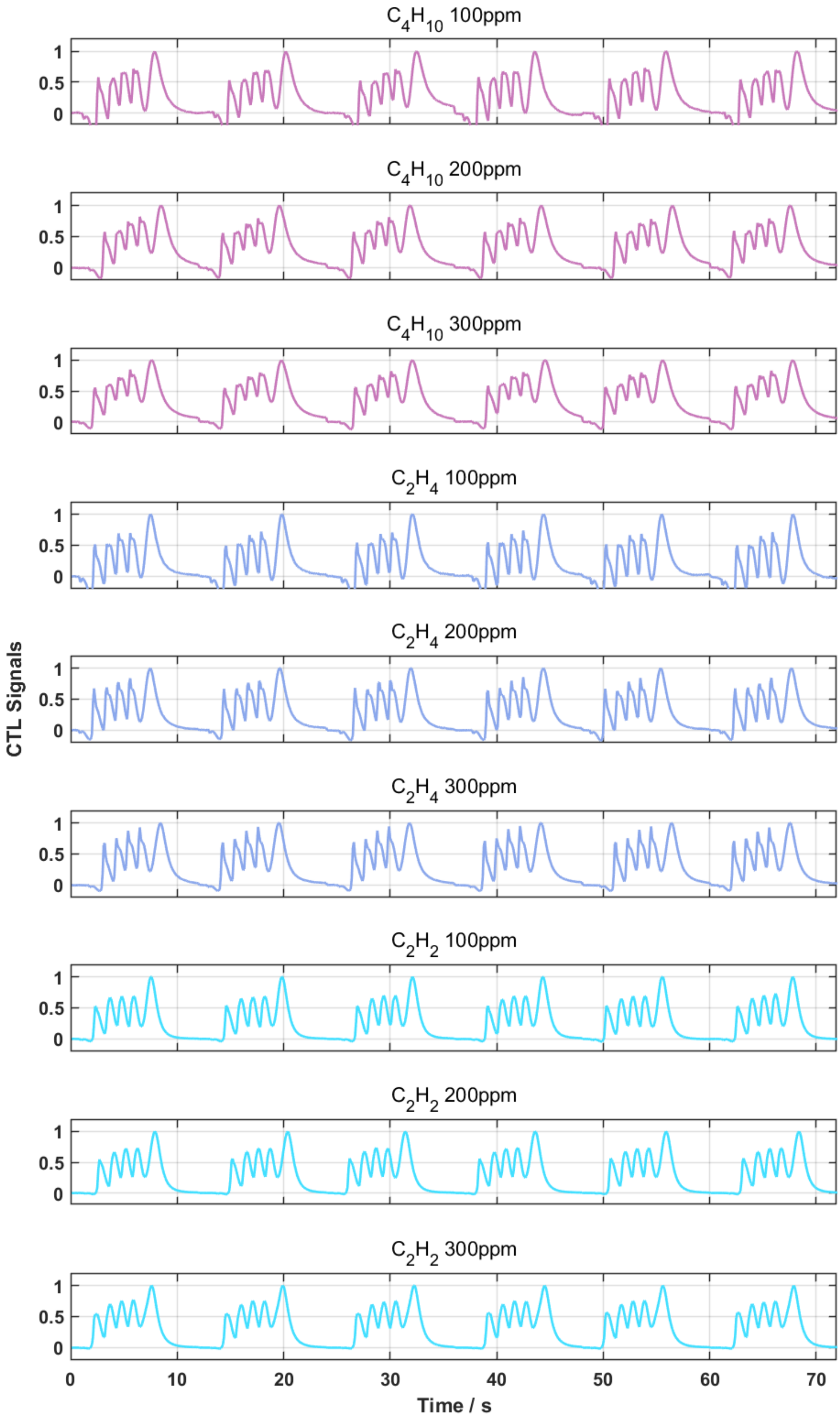

Fig. S7.

Normalized signal examples of 9 gas samples measured with the MOSS strategy. The differences between various VOC species can be observed from the peak distribution of normalized signals. Visually, the middle peak of ethylene appears higher and sharper, while acetylene's middle peaks are relatively more evenly distributed. The data used for classification can be referred to in the PCA diagram presented in the main text.

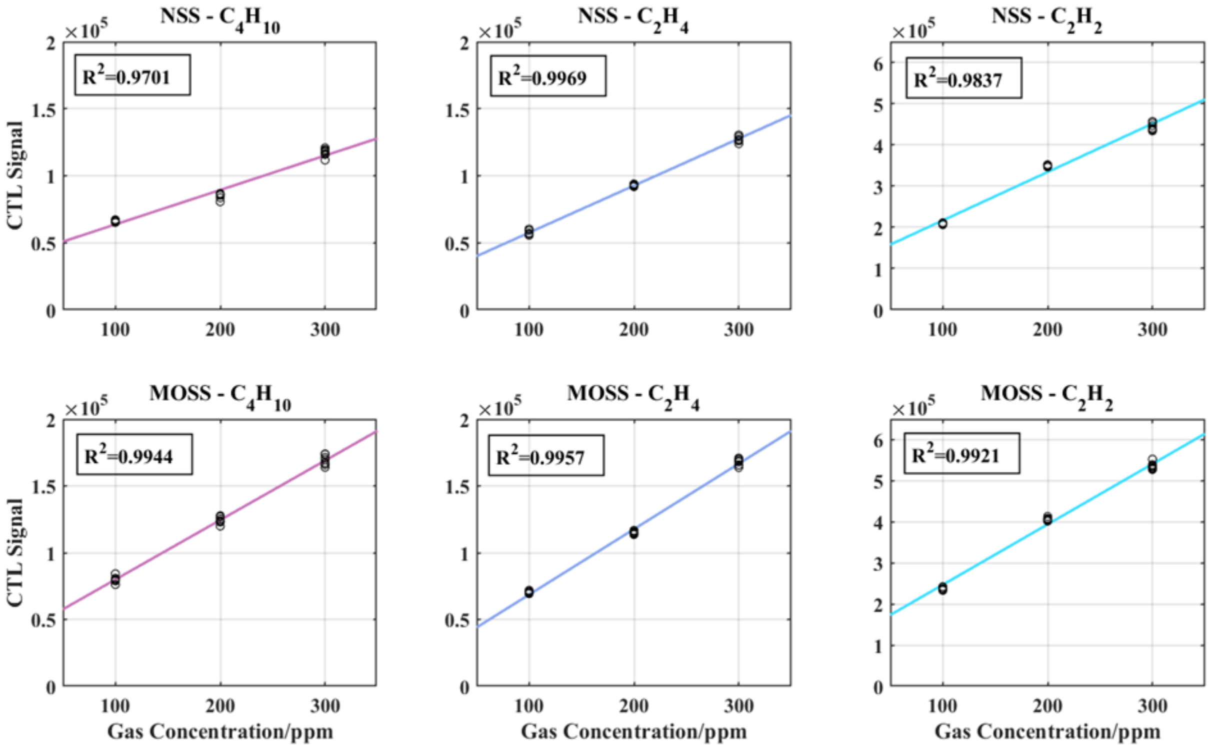

Fig. S8.

Linear fit results of the peak values of the single CTL sensor to the measured VOC concentrations. The signal with the NSS strategy has only one peak, while the signal with the MOSS strategy has five peaks. And the last peak value is uniformly taken as the dependent variable for the linear fit. It can be seen that the peak values are well linear with measured VOC concentrations, even for different VOC species. This also demonstrates that the MOSS strategy allows a single CTL sensor to recognize both the concentration and species of VOC gas via only one measurement.

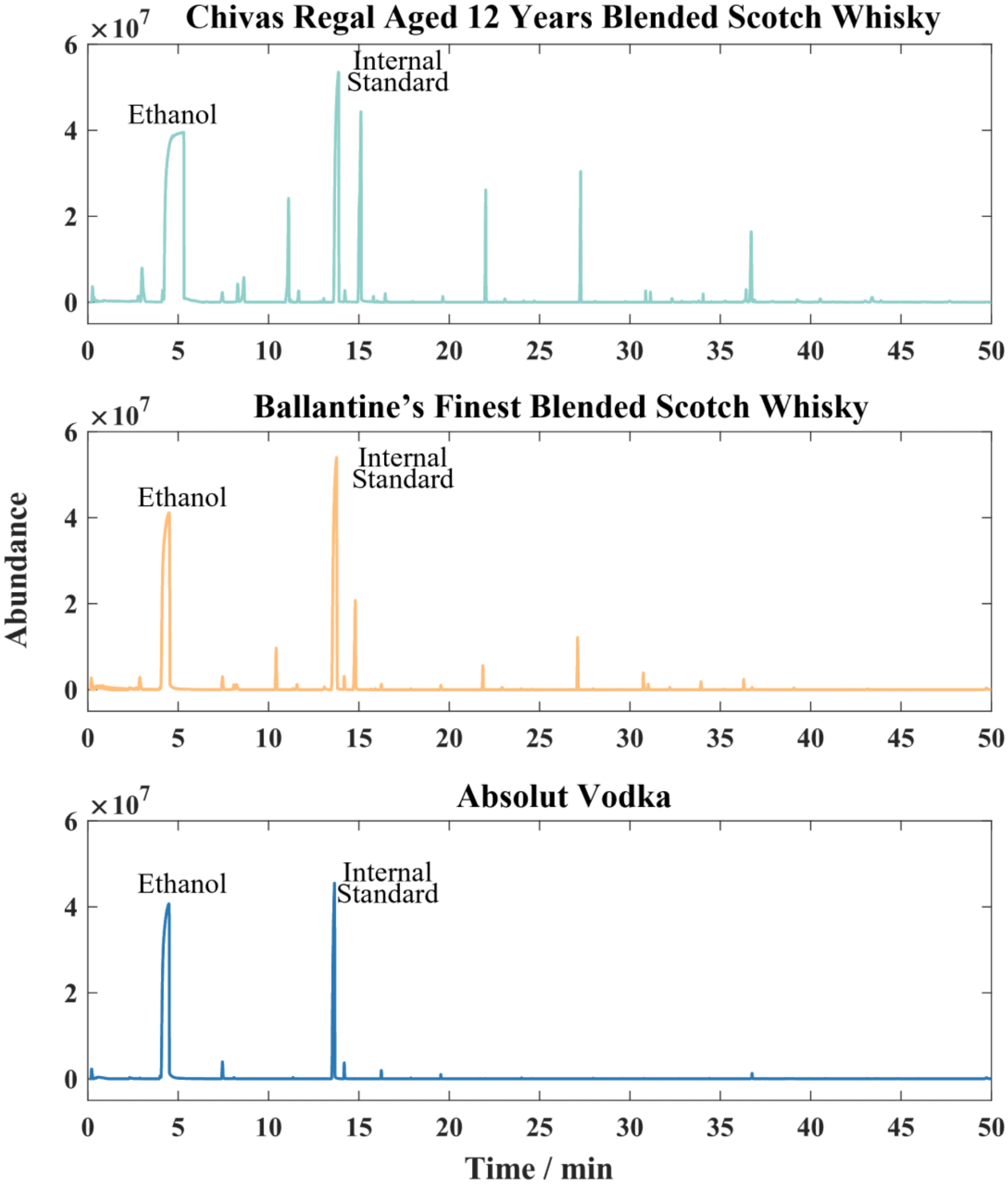

Fig. S9.

Spectrums of Gas Chromatography-Mass Spectrometry (GC-MS) of three liquors (W1, W2 and V). The standard solution of pentyl acetate is used as the internal standard to compare the composition of different liquors, and the concentration of pentyl acetate in the measured liquor sample is 438 μg/ml. It can be seen that ethanol is the main organic component of all liquors, with trace differences of other aroma compounds in different liquors. For most gas sensors, the presence of ethanol is the main obstacle that prevents them from recognizing different liquors.

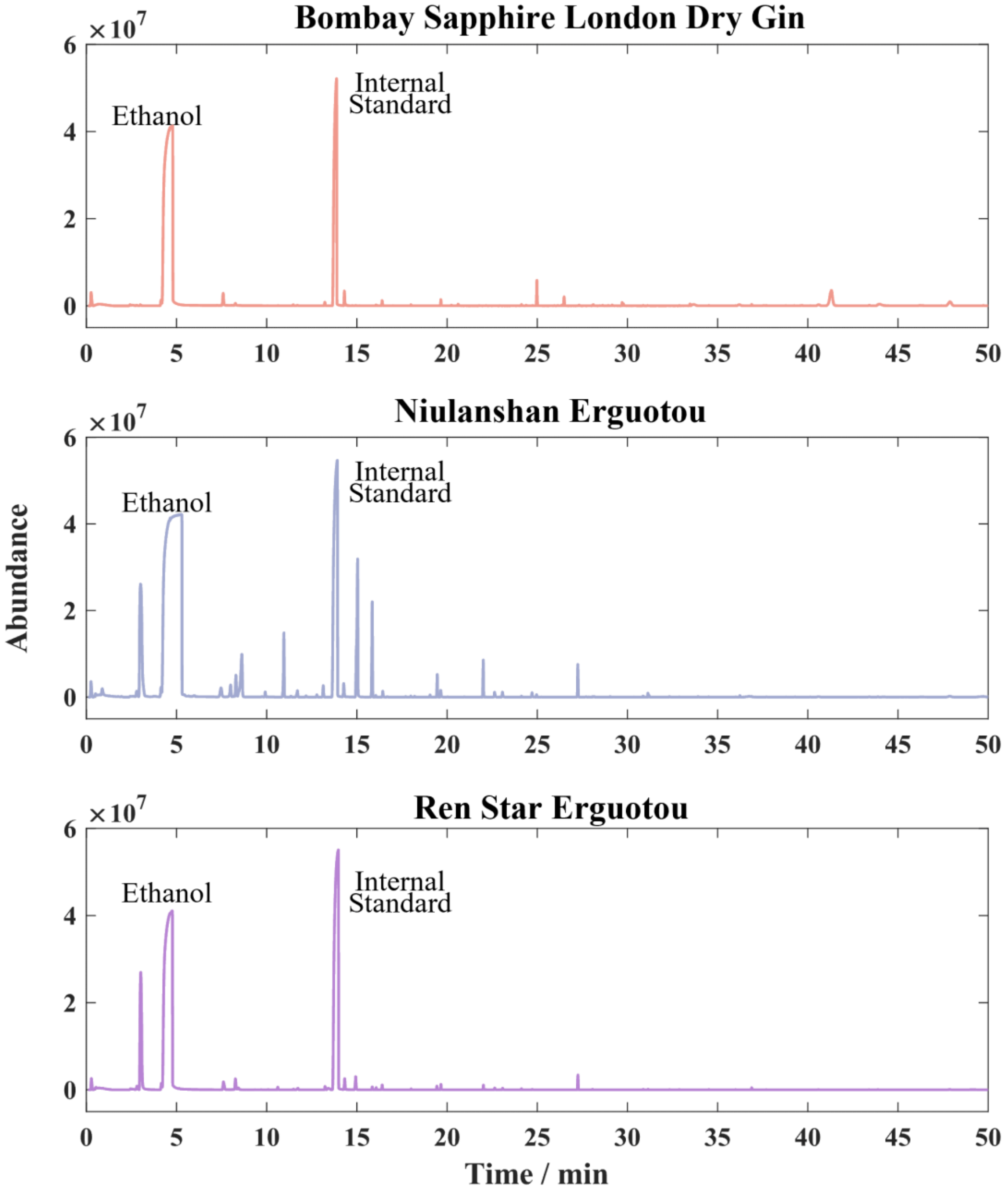

Fig. S10.

Spectrums of Gas Chromatography-Mass Spectrometry (GC-MS) of three liquors (G, E1 and E2).

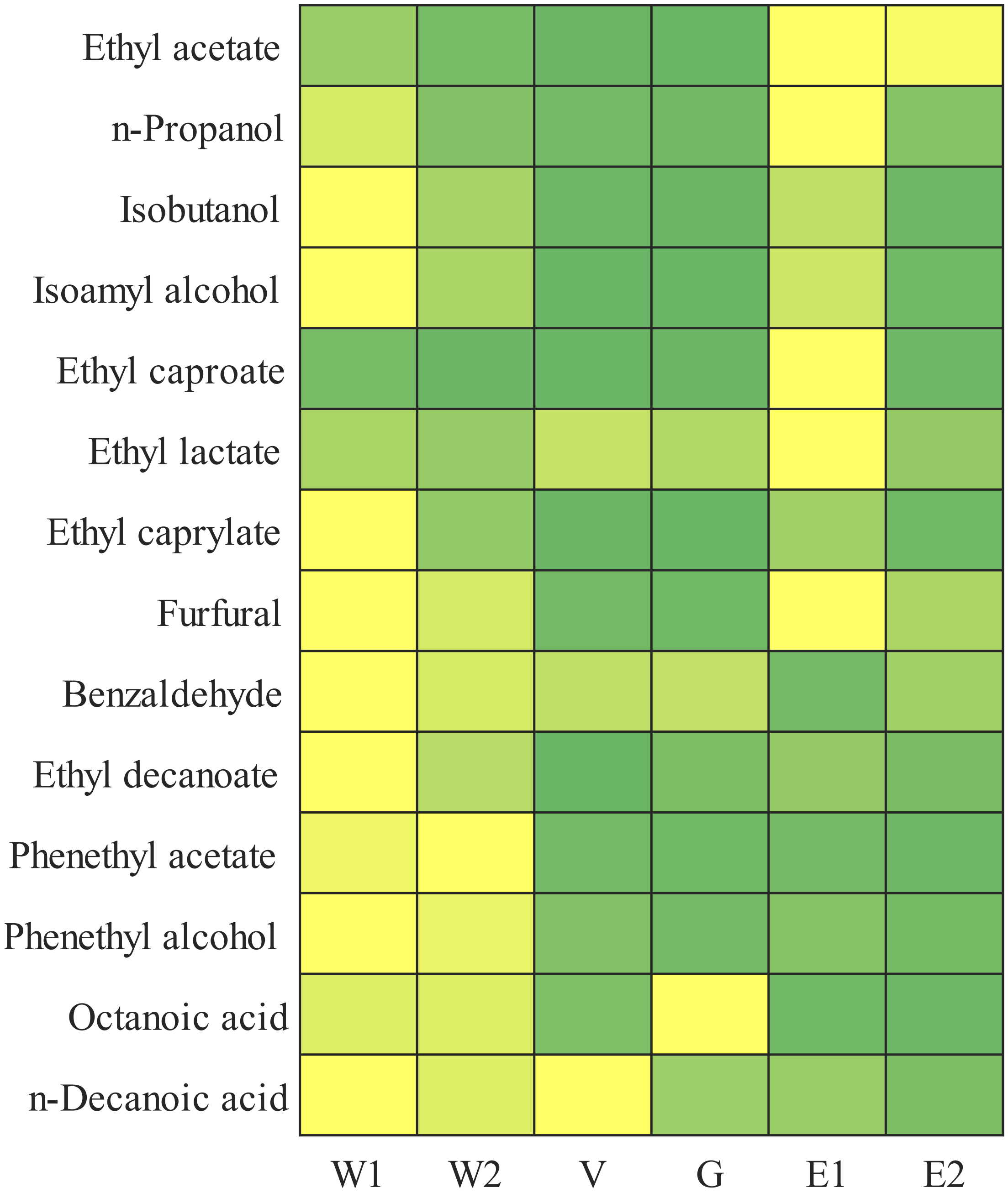

Fig. S11.

Relative comparison of the main aroma compound content in 6 liquors. Based on the GC-MS results, the contents of several aroma compounds commonly found in liquors, namely alcohols, esters, acids, and aldehydes, are quantified. And the differences of aroma compounds lead to the liquor recognition by electronic noses. It should be mentioned that the concentrations of all aroma compounds in this Figure are **no more than 150 μg/ml** which is so low that it is difficult for an untrained person or a common gas sensor to recognize liquors.

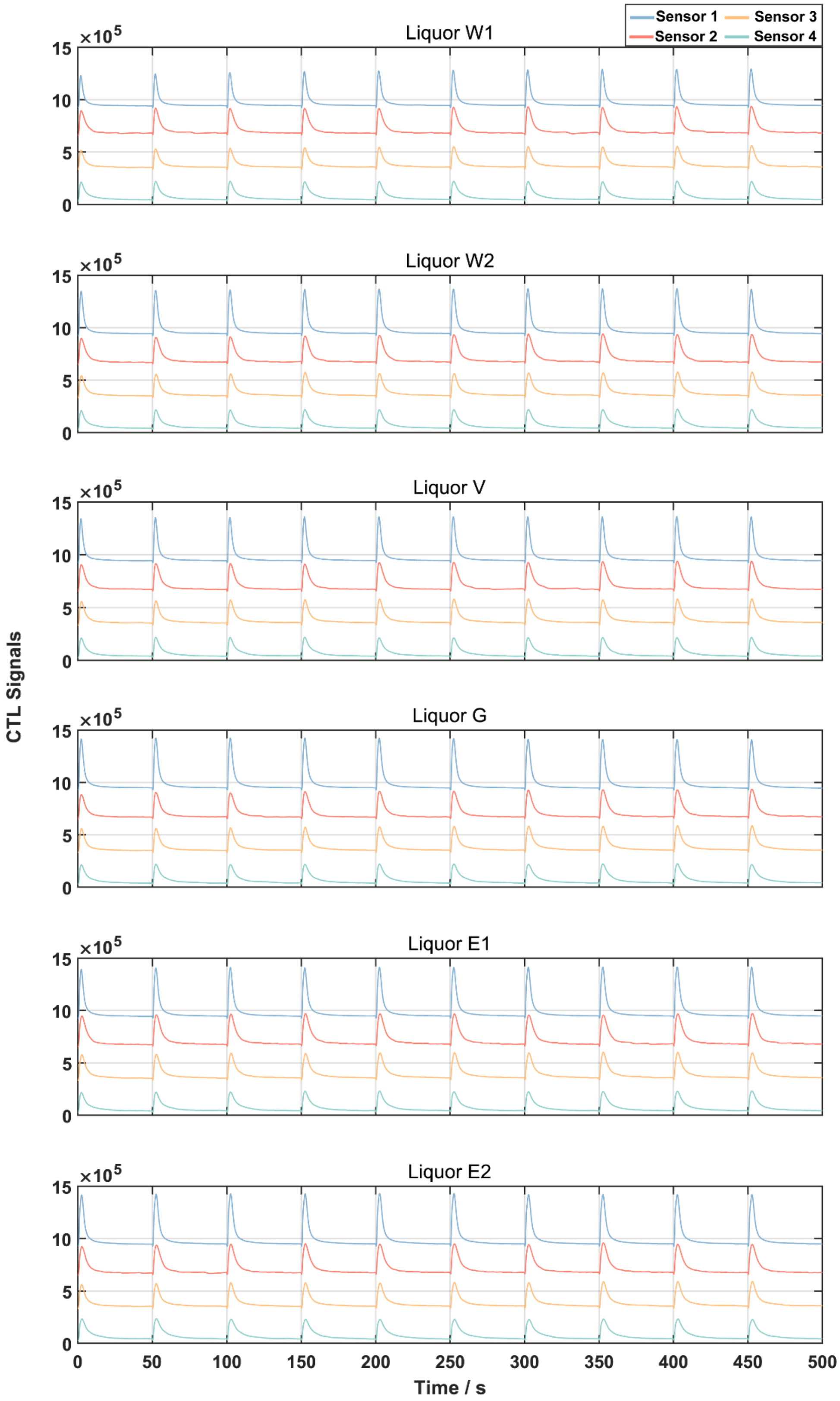

Fig. S12.

Signal examples of 6 liquors measured with the NSS strategy. It can be observed that there is good consistency in the testing signals for each liquor. The testing for each gas was repeated 12 times. In this figure, to showcase the details of the signal waveform, 6 groups of signals are displayed.

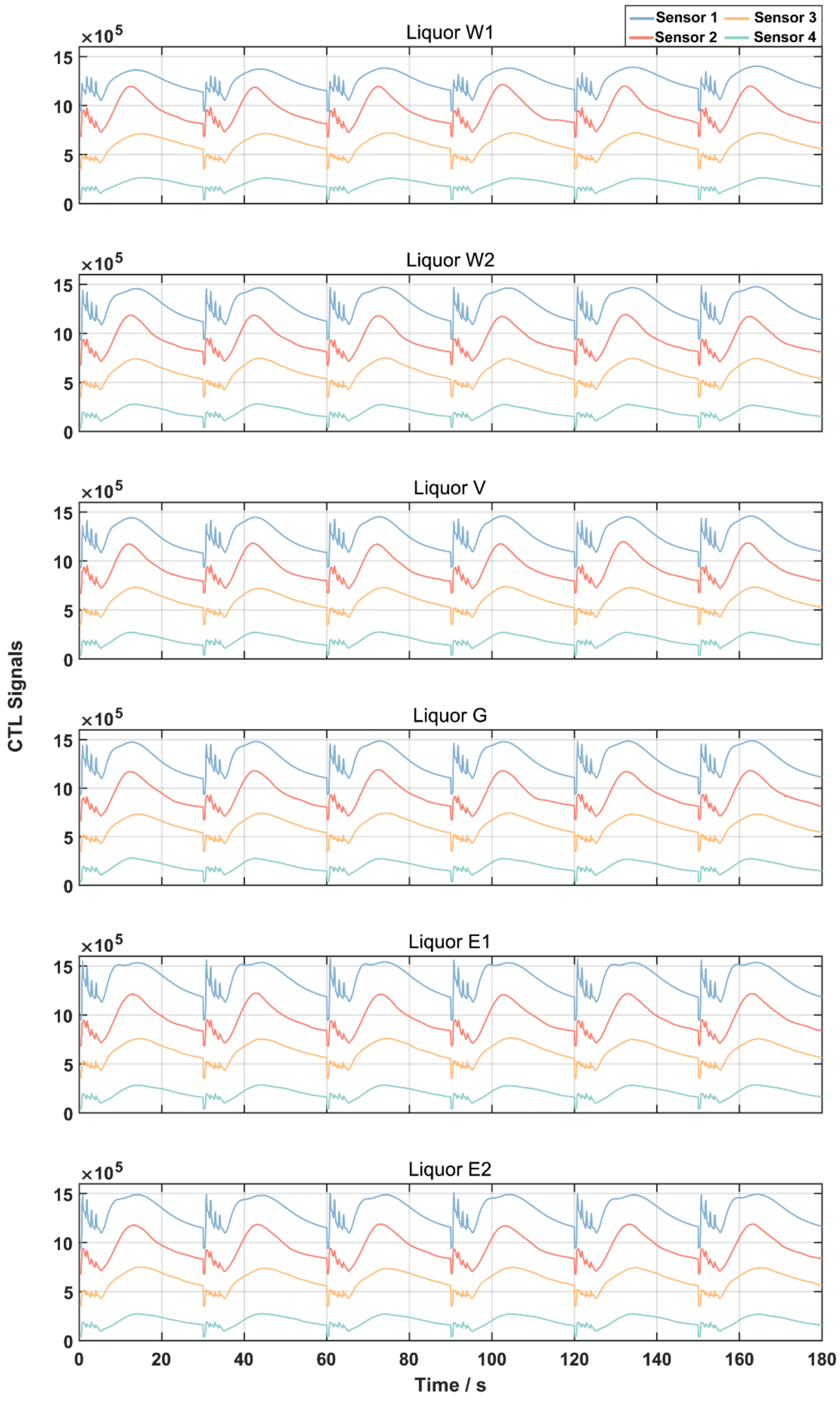

Fig. S13.

Signal examples of 6 liquors measured with the MOSS strategy. It is evident that there is a high level of consistency in the testing signals for each liquor. The testing for each gas was conducted 24 times. Furthermore, the test was repeated over the course of two days, and the data demonstrated excellent reproducibility.

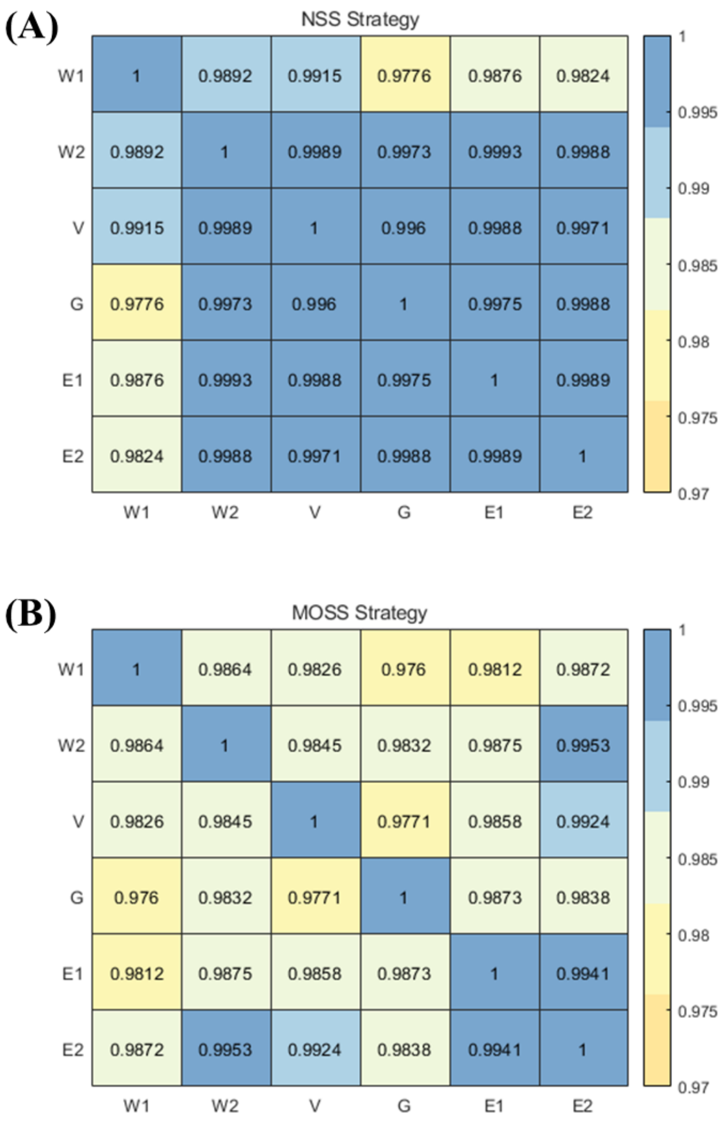

Fig. S14.

The correlation coefficients of signal features in the liquor recognition experiments. For both the NSS and MOSS strategies, measurement signals from six types of liquors are selected for comparison. From these signals, a set of peaks and troughs were manually extracted as feature vectors, following the method outlined in the paper. By comparing the correlation coefficients between these vectors, it can be observed that, overall, the MOSS strategy is capable of reducing the correlation between feature vectors, thus enabling better differentiation between different types of liquors. It should be noted that this is only a comparison of one set of measurement signals. The complete predictive performance should be evaluated based on the 5-fold cross-validation experiments outlined in the paper.

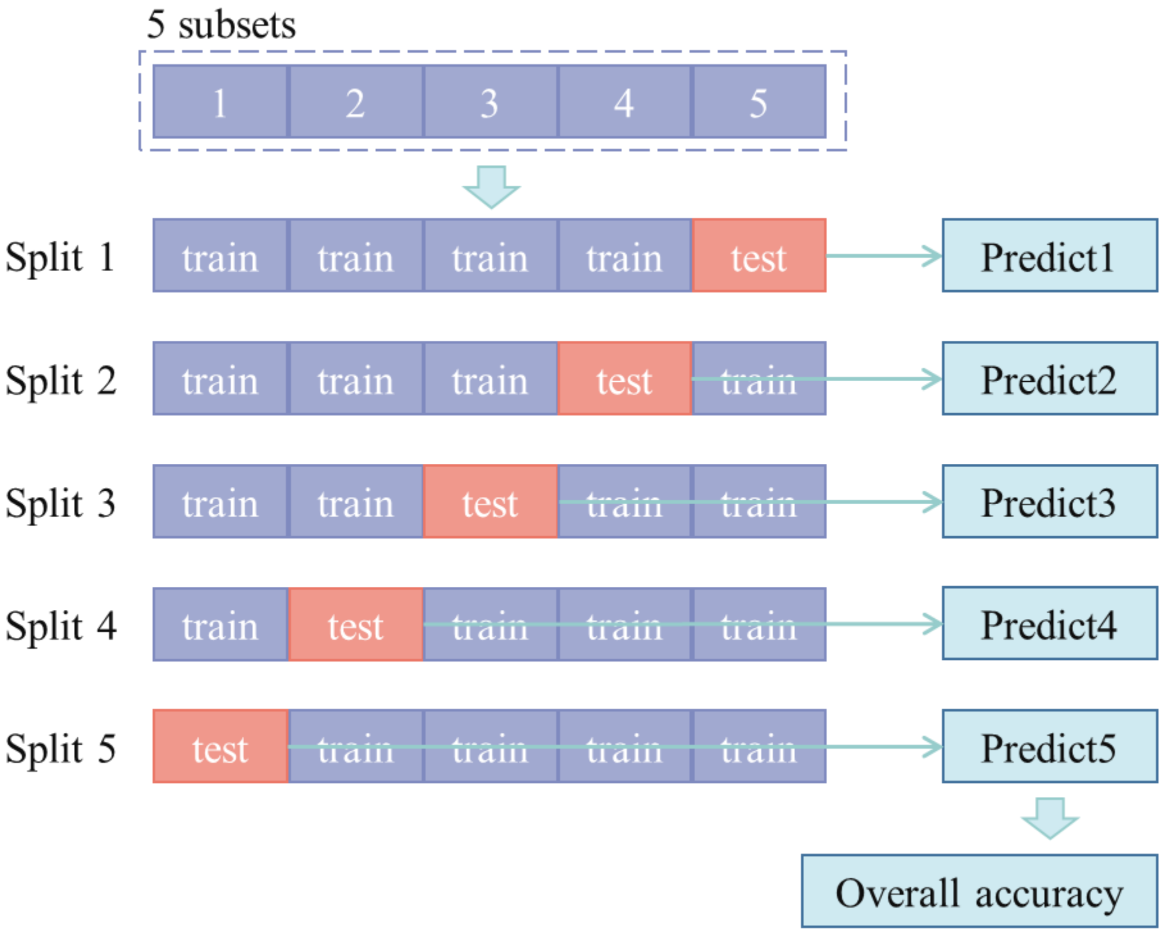

Fig. S15.

Detailed description of 5-fold cross validation experiment. This figure shows that the total dataset is equally split into 5 independent subsets each of which contains all kinds of liquor samples. And the SVM is trained and tested for 5 times in which one subset is used as the test set and other 4 subsets are integrated into the training set. Consequently, every sample in the total dataset is predicted once, and the overall accuracy can be calculated.

Carrier Gas Rate	$0.5(\pm 0.005)\ slm$
Temperature	$280\ (\pm 1)$ ℃
Injection Volume	$10\ mL$
Maximum Injection Rate	$1\ slm$
PMT Sample Time	$0.1\ s$
Sensor Chamber Size	$\phi 20 * 50mm$
Air Path Size	$\phi 3.5 * 800mm$

Table S1. Detailed experiment parameters for CTL sensing.

Movie S1.
The computational fluid dynamics simulation of Multiple Overlapping Sniffs (MOSS) strategy.
